# Assessing Trends in the Desire to Avoid Pregnancy: A Cautionary Note

**DOI:** 10.1111/sifp.12270

**Published:** 2024-07-23

**Authors:** John B. Casterline, Laila El-Zeini, Mobolaji Ibitoye

**Affiliations:** Institute for Population Research, Ohio State University, Columbus OH 43210, USA.; Department of Statistics, Faculty of Economics and Political Science, Cairo University, Dokki, Giza 12311, Egypt.; Department of Urban-Global Public Health, Rutgers University, Newark NJ 07102 USA.

## Abstract

The desire to avoid pregnancy—to delay the next birth or have no further births—is a fundamental sexual and reproductive health indicator. We show that two readily available measures—prospective fertility preferences and the demand for contraception [Demand] construct—provide substantially different portraits of historical trends. They also yield correspondingly different assessments of the sources of contraceptive change. We argue, with supporting empirical evidence, that Demand enormously overstates the historical trend in the desire to avoid pregnancy because Demand as currently constructed is in part a function of contraceptive prevalence. This makes for “reverse causality” in decompositions of contraceptive change, producing an upward distortion on the order of 25 percentage points in the amount of contraceptive change attributed to a change in fertility desires. Decomposition of contraception change free of the distortion reveals that contraceptive change has been due almost entirely to more complete implementation of fertility preferences. This is explained in part by the surprisingly slight historical change in preferences, a fact we document and then show is a consequence of a historical shift in parity composition toward lower parities.

## INTRODUCTION

Conditioning contraceptive prevalence on fertility preferences is now a well-established practice in international sexual and reproductive health (SRH) research. While the concepts of “KAP (knowledge, attitudes, and practice)-gap” and “unmet need for contraception” precede the 1994 International Conference on Population and Development, the historic pivot at that conference away from demographic targets toward the goal of satisfying the reproductive aspirations of women and couples made it imperative to employ indicators consistent with that goal. Initially, unmet need served as the focal indicator, but by the mid-2010s unmet need was supplanted by *Demand* and *Demand Satisfied* (Westoff 2006; Choi et al. 2015). The former is defined as the proportion of women of reproductive age who wish to avoid pregnancy, while the latter is the proportion of this subgroup who are using contraception. These are cross-sectional indicators that provide a snapshot of a historical moment.

*Demand* is conventionally measured as the sum of the proportion of women using contraception and the proportion of women with “unmet need for contraception,” both confined to women in stable unions (formal or informal). “Unmet need for contraception” is constructed following an algorithm developed by the Demographic and Health Surveys (DHS; Bradley et al. 2012). This measure is rather complicated to construct, but it is available in DHS data files and the DHS has also posted code for its construction. *Demand Satisfied* is defined as the proportion of women using contraception among women in the *Demand* group.

Notwithstanding its popularity, the construct *Demand* has been subjected to much criticism over the past two decades. For one thing, *Demand* does not indicate an explicit desire to practice contraception, instead this desire is inferred either from behavior (current contraceptive use, a result of past or recent decisions) or from stated fertility preferences. Scholars have noted that a preference to delay the next birth or have no further births is not tantamount to a desire to practice contraception (Westoff 2012; Cleland, Harbison, and Shah 2014; Senderowicz and Maloney 2022; Senderowicz et al. 2023). Developing more direct measurements of the desire to practice contraception is an active area of work (Aiken et al. 2016; Senderowicz 2020; Miller, de Paula, and Valente 2020; Speizer, Bremner, and Farid 2022; Fabic 2022; Bornstein et al. 2023). In the meantime, *Demand* and *Demand Satisfied*, with women’s fertility preferences as a foundation, remain core SRH indicators. Notably, *Demand Satisfied* is an SDG (Sustainable Development Goals) indicator—indicator 3.7.1 under Target 3.7 “Universal access to sexual and reproductive care, family planning and education.” It is one of only two SRH indicators (the other concerns adolescent fertility) among a total of 231 SDG indicators. Hence, regular assessment of levels and trends in *Demand* and *Demand Satisfied* is a high priority (United Nations Population Division 2022).

In this note, we show that *Demand*—a composite of contraceptive prevalence and “unmet need for contraception”—provides a markedly different portrait of trends in the desire to avoid pregnancy than one obtains from fertility preferences alone. We then document how these discrepant portraits of trend yield large differences in assessments of the contribution of change in fertility preference to contraceptive change. We also show how the measurement rules adopted in the conventional construction of *Demand* contribute to these documented discrepancies. Finally, we explain why trends in fertility preferences—the fraction of in-union women who want to avoid pregnancy—are so slight, an intriguing and not widely recognized empirical fact.

## CONSTRUCTING AN INDICATOR OF *DEMAND*

In the research literature of the past five decades, the most common empirical basis for estimates of the prevalence of a desire to avoid pregnancy has been the prospective preference survey item. With slight variations in wording, this is the question “do you want another child?” Among attitudinal items on fertility desires, this item has been shown to have the highest validity and reliability, according to various criteria (Casterline and El-Zeini 2007; Cleland, Machiyama, and Casterline 2020). A follow-up item asks about desired timing of the next birth among those who want another birth. Convention in recent decades has been to classify women who state a desire to have the next birth at least two years in the future as having a desire to delay. Hence, the desire to avoid pregnancy consists of two categories of women: those who want no more children and those who want to delay two or more years. One can assess trends in the desire to avoid pregnancy by simply tracking over time the cross-sectional prevalence of women falling into one of these two categories.

The established construction of *Demand* departs from this approach. As noted above, *Demand* is conventionally measured as the sum of the proportion of women using contraception and the proportion of women with “unmet need for contraception,” both confined to women in stable unions (formal or informal). The “unmet need for contraception” algorithm relies on prospective preferences for the majority of women, but for women who at the survey are pregnant or in postpartum amenorrhea (0–23 months postpartum) the algorithm makes use of the wanted status of the current pregnancy or the most recent birth (Bradley et al. 2012). The wanted-status categories are “wanted, on time,” “wanted, too soon,” and “not wanted.” Women in the latter two categories are classified as having unmet need (for spacing and for limiting, respectively). The attitudinal foundation for *Demand* is, therefore, a composite of prospective preferences and retrospective preferences. The logic of the unmet need algorithm, as first proposed by Westoff and Ochoa (1991), is sound. But the validity of the resulting estimates of the desire to avoid pregnancy is jeopardized by retrospective underenumeration of births/pregnancies that occurred “too soon” or were “not wanted” (Bankole and Westoff 1998; Casterline, El-Zanaty, and El-Zeini 2003; Koenig et al. 2006; Casterline and El-Zeini 2007; Jain et al. 2014; Cleland, Machiyama, and Casterline 2020).

There are two further features of *Demand* that depart from sole reliance on prospective preferences. First, contraceptive users include some women who indicate a desire to have their next child within two years. Because these women are using contraception, by definition they are assumed to have *Demand*. But were they not using it, they would not be classified as having unmet need (and hence would not contribute to *Demand*). This is, on the face of it, inconsistent treatment of women who wish to have a child relatively soon. Second, the unmet need algorithm sets aside women who are infecund as determined by several different criteria (Bradley et al. 2012). But if they are using contraception, they are assumed to have *Demand*, another inconsistency.

Falling between relying entirely either on prospective fertility preferences or on *Demand* are possible measures of the desire to avoid pregnancy (desire to delay and to stop combined) that are based to varying extents on prospective preferences and the retrospective wanted status of the current pregnancy or most recent birth. Six such variants of the measures are specified in Table 1. These are as follows:

- Prospective preferences for all currently-in-union women; for currently pregnant women, this is what they want after the birth of the child;
- Prospective preferences except for currently pregnant women, for whom wanted status of the pregnancy is used;
- Prospective preferences except for currently pregnant women and postpartum amenorrheic women, for whom wanted status of current pregnancy/last birth is used;
- Variant C, except exclude women identified as infecund using the DHS algorithm;
- Unmet need variable—a composite of prospective preferences and wanted status of pregnancy/birth, constructed according to DHS algorithm (which uses the prospective preferences of the postpartum amenorrheic women if they are users and excludes nonusers identified as infecund);
- *Demand* as standardly constructed (i.e., contraceptive prevalence + unmet need).

The six variants progress from depending entirely on the prospective preference item to gradually introducing different elements from the *Demand* construct. We use this gradual transition to investigate the effects of different measurement choices.

Suppose one regards prospective preferences as offering the most straightforward foundation for assessing the prevalence of the desire to avoid pregnancy; how much does the portrait provided by the alternative measures differ? A great deal, as it turns out, as shown in Table 2. Variants A and B, with the strongest dependence on prospective preference, produce the highest prevalence of the desire to avoid pregnancy. This prevalence is markedly lower—about 10 percentage points lower—when the fertility preference of postpartum amenorrheic women is with reference to their most recent birth (variants C–F). The differences among the four variants that make use of retrospective preferences (Variants C–F) are small. A further pattern in Table 2 is the larger difference in sub-Saharan Africa (SSA) between Variants A–B and Variants C–F, that is, between variants based entirely/mainly on prospective preferences and variants that rely substantially on retrospective preferences. This follows from the fact that postpartum amenorrheic women constitute a larger fraction of women in SSA.

In this note, our focus is on trends, for which the choice of measure is if anything more consequential than the effects on prevalence examined in Table 2. Table 3 shows trends in the six variants. The trends are expressed as percentage point increase per decade among 53 countries and have been obtained via survey-level linear regression with fixed effect for each country; in effect, Table 3 presents the average of country-by-country regressions.

The extremes are represented by Variants A and F, that is, pure prospective preferences versus *Demand*, with the other variants falling somewhere in between. The discrepancy between A and F is large: for all regions, whereas Variant A increases by less than one percentage point per decade on average, Variant F (*Demand*) increases by 4.2 percentage points per decade on average, a far steeper trend. (Below we examine more closely the surprisingly slight trend in prospective preferences, i.e., Variant A.) The large discrepancy applies to both SSA and other regions, although it is somewhat larger in other regions. Each step between Variant A and Variant F is accompanied by an increase in the steepness of the trend toward a higher fraction of women desiring to prevent pregnancy.

## SOURCES OF CONTRACEPTIVE CHANGE

To what extent can the increase in contraceptive prevalence be attributed to changes in fertility preferences? For many decades, this has been a central question in scholarly debates about contemporary fertility declines (Pritchett 1994; Bongaarts 1997; Lam 2011; Günther and Harttgen 2016; Bongaarts and Hodgson 2022), with direct implications for population policies and programs. We demonstrate here that the answer to this question hinges on which of the variants of the desire to avoid pregnancy is employed.

The question implies a decomposition in which contraceptive change is attributed to each of two components:

- change in the prevalence of the desire to avoid pregnancy and
- change in rates of contraceptive use among women who desire to avoid pregnancy, that is, change in the implementation of preferences.

In the standard terminology of demographic decomposition, these are “composition” and “rate” components, respectively (Das Gupta 1993).

Decompositions can be performed using any one of Variants B–F of the desire to avoid pregnancy.^1^ In brief, using national demographic survey data, women are stratified according to their desire to avoid pregnancy, with rates of contraceptive use calculated for each stratum. This allows for a standard demographic decomposition based on the equation^2^:

(1) $$U=\sum c_{p}r_{p,}$$

where U is contraceptive prevalence (percentage of in-union women using a method), c is composition: the percentage of in-union women in each preference category, r is the rate: the proportion using in each preference category, and p denotes a preference category.

In Variants B–E, there are three preference categories: soon, later, and no more. Variant F distinguishes only between two groups: women identified to have *Demand* and those who do not. By convention, all contraceptive users are considered to have *Demand*. This is a distinct and, as we will shortly demonstrate, consequential feature of Variant F. In contrast to the other variants, the rate component ($r_{p}$) is nonzero for only one preference stratum (those who have *Demand*). As a result, the decomposition that employs Variant F (*Demand*) can be simplified to a more concise expression:

(2) U=D*DS,

where U is contraceptive prevalence (percentage of in-union women using contraceptives), D is *Demand*: percentage of unmet need + percentage of contraceptive prevalence, and DS is *Demand Satisfied*: used as a proportion of *Demand* (U/D).

Are the results of such decompositions of changes in contraceptive use sensitive to how the need or desire for such use is operationalized? As it turns out, the results and their implications about the sources of contraceptive change are substantially different, especially if one compares Variant B (reliance on prospective preferences with the exception of pregnant women) and Variant F (reliance on the conventional *Demand* construct).

The differences between the five variants are documented in Table 4. The data are from DHS from the early 1990s to the present. Sixty-five decompositions are conducted (37 SSA, 28 other regions).^3^ If one relies on Variant B—heaviest dependence on prospective preferences—the median percentage of contraceptive change attributed to preference change is 3 percent. By contrast, if one relies on Variant F—*Demand* construct—the median is 28 percent. That is, the difference between the two variants in the fraction of contraceptive change attributed to preference change is 25 percentage points. According to Variant B, the contribution of preference change is inconsequential; by contrast, according to Variant F, the contribution of preference change is substantial (though still well below one-half).

The decompositions in Table 4 are carried out using DHS surveys only. Bongaarts and Hodgson (2022) have performed a more encompassing decomposition exercise using the United Nations Population Division estimates of *Demand* and *Demand Satisfied* for all low- and middle-income countries for the period 1970–2015 (Bongaarts and Hodgson 2022; United Nations Population Division 2022).^4^ They report average contributions of preference change of 31 percent in SSA, 38 percent in Asia and North Africa, and 39 percent in Latin America. Note that in those regions where the decompositions encompass a substantial increase in contraception (Asia, North Africa, Latin America), Bongaarts and Hodgson conclude that nearly 40 percent of contraceptive increase can be attributed to a change in the prevalence of the desire to avoid pregnancy, whereas Variant B in Table 4 (relying mainly on prospective preferences) yields an average of 0 percent in the non-SSA regions.

## WHY THE DIFFERENT TRENDS?

The marked discrepancy in historical trends evident in Table 3, and the difference in decomposition results evident in Table 4 can be explained by the differential treatment across the variants of three subgroups of women: (1) women who “want soon”; (2) women who are postpartum amenorrheic; (3) women classified as infecund. For each group, their treatment under the *Demand* construct departs from sole reliance on prospective fertility preferences. Of special concern is the fact that the treatment of these three subgroups of women according to the *Demand* construct induces spurious “reverse causality” between trends in fertility preference and contraceptive use: specifically, an increase in contraception per se generates the appearance of an increase in the desire to avoid pregnancy due to measurement convention rather than genuine change in desires.

We consider each subgroup of women in turn, with a focus on the implications of how they are treated in the construction of *Demand*.

### Women Who “Want Soon”

As noted above, the treatment of this sub-group of women under the *Demand* construct is inconsistent: these women do not contribute to *Demand* if they are not using contraception, but they do contribute to *Demand* if they are using. Put otherwise, “want soon” women cannot have unmet need, though they can have met need.

The consequence of this measurement idiosyncrasy is that increased rates of use among this subset of women in itself produce an increase in *Demand*. A decomposition utilizing Variant F (*Demand*), hence, treats increased rates of contraceptive use within a preference stratum as a change in composition (a shift into *Demand*).^5^

The distortion this produces is illustrated in Table 5. This is a fictional illustration in which contraceptive prevalence in the first survey is 41 percent and in the second survey is 53 percent. Preference composition is precisely the same in both surveys; the 12 percentage-point increase in contraception is due entirely to increased rates of use in each preference category.^6^ Accordingly, decompositions employing Variants B–E attribute none of the increase in contraception to preference change (“composition”). But if one employs Variant F (*Demand*), 12 percent of the increase in contraception is attributed to a change in composition (as determined by *Demand*) even though the underlying preference composition has not changed.

It is worth pointing out that such inconsistent treatment of women who report a desire to have births soon (within two years) is due to an underlying assumption that any woman using contraception wishes to avoid pregnancy and hence truly has need for contraception. The intensive empirical analysis of Canning and Karra (2023), focusing on women who want a child soon but are using contraception, raises serious doubts about this assumption. Factors such as coercion and the use of condoms to prevent sexually transmitted diseases could explain this apparent misalignment between stated preferences and use. In addition, the choice of a two-year window for “soon” is arbitrary and could well include women whose wish is to avoid pregnancy for the time being. While such imprecision is a measurement concern that bears on all analyses of the relationship between fertility preferences and use, it becomes especially problematic when the mere use of contraception is used to infer preferences.

### Women Who Are Postpartum Amenorrheic

In Variants C and D, the preference status of all postpartum amenorrheic women (through 23 months postpartum) is with reference to their previous, rather than future, pregnancy. The rationale is that these women are not at risk of getting pregnant at the survey date and hence have no reason to use contraception.

Variants E and F, adopting the conventional construction of *Demand*, treat postpartum amenorrheic women differently depending on whether or not they are using contraception. If they are using, they have *Demand* regardless of their prospective (or retrospective) preferences. If they are not using, their preference category depends on their report of the wantedness of the last birth, with those reporting their births as “wanted, too soon” or “not wanted” classified as having *Demand*. This produces a net upward shift into *Demand*, even without any change in reported attitudes (prospective or retrospective), whenever there is an increase in contraceptive use among women who reported their last birth as “wanted, on time.” This is highly likely to occur, to a lesser or greater extent, especially because of the response bias toward “on time” in retrospective reports (see citations above, including a recent review in Cleland, Machiyama, and Casterline 2020, of the empirical evidence suggesting response bias of this sort).

This problem can be illustrated through two hypothetical women both of whom are six months postpartum, wish to postpone their next births, and state that their last births were “wanted, on time.” Suppose that one of the two women is not using contraception while the other is using it. Although the two women have exactly the same preferences (both prospective and retrospective), one of them is classified as lacking *Demand*, while the other is classified as having *Demand*. As contraception becomes more widespread, this differential treatment of users itself yields an increase in *Demand*. This in turn inflates the amount of contraceptive change attributed to compositional change in a decomposition that employs Variant F and Equation (2).

### Women Classified as Infecund

The DHS unmet need algorithm starts by setting aside women reported to be currently using contraceptives. All users, regardless of their fertility preference or any pregnancy risk, are considered to have met need. Nonusers, on the other hand, are classified into different categories and their need for contraception is based on the risk of becoming pregnant (sexual activity, fecundity status) as well as fertility preference. If a woman is determined to be infecund, she does not have unmet need and hence does not contribute to *Demand*.^7^ But contraceptive users who otherwise would have been classified as infecund do contribute to *Demand*. This inconsistency is analogous to the treatment of women who “want soon” discussed above. In both cases, a subset of women is classified as having *Demand* (or not) based on their contraceptive use. Ceteris paribus as contraceptive prevalence rises, *Demand* increases.

## INCREASE IN *DEMAND* DUE TO MEASUREMENT CONVENTION

The feature that unifies the treatments under the *Demand* construct of these three distinct subgroups of women is as follows: an increase in contraceptive use itself generates an appearance of an increase in the desire to avoid pregnancy, simply due to measurement convention (rather than true attitudinal change). That is, the apparent change in the desire to avoid pregnancy is an artifact of measurement. And because the measurement artifact is tied to contraceptive use, assessments of the contribution to contraceptive change of the desire to avoid pregnancy are unavoidably distorted.

A closer examination of Table 4 suggests how much distortion is due to the inconsistent treatment of each of these subgroups. The magnitude of the distortion each one produces can be judged by the following comparisons:

F versus E: 1. Treatment of “want soon” women

E versus D: 2. Treatment of postpartum amenorrheic women

D versus C: 3. Treatment of infecund women

Judging from Table 4, the largest distortion in decomposition results from the treatment of “want soon” women (F vs. E). The impact of the treatment of postpartum amenorrheic women (E vs. D) is especially large in SSA where durations of postpartum amenorrhea are longer, while the classification of infecund women (D vs. C) is more impactful in other regions.

Note that even Variant A—prospective preferences alone—suffers from the “reverse causality” identified here, that is, an increase in contraceptive use itself producing an impression of an increase in the desire to avoid pregnancy. In the DHS, women who are sterilized for contraceptive purposes are assumed to want no additional children. But this does not follow necessarily. Some sterilized women may, at the time of the survey, wish to have further children (although this is physiologically impossible).^8^ It is safe to assume that the distortion this produces is small in most countries except where there has been a sharp trend toward a higher prevalence of sterilization.

## HOW TO EXPLAIN THE SLIGHT TREND IN PREFERENCES?

A surprise in the empirical results in Table 3 is the slow historical trend in fertility preferences (desire to delay or stop): 0.3 percentage points per decade, almost no trend at all. This in turn helps explain the very small contribution of preference change in the decomposition results in Table 4 (Variant B). Declines in the ideal number of children have been documented (Westoff and Bankole 2000; Westoff 2010), as have corresponding increases in the parity-specific desire to have no further children (Westoff 2010; Casterline and Agyei-Mensah 2017). But with the exception of our own recent scholarship (Ibitoye, Casterline, and Zhang 2022), we do not find in past scholarship a recognition that preferences among all women of reproductive age (i.e., pooled across parity) have changed slowly over time. For one, Bongaarts over the decades has asserted the opposite, namely that fertility decline is accompanied by a surge in the fraction of women who wish to terminate childbearing (Bongaarts 1992; 1997; Bongaarts and Hodgson 2022; Bongaarts et al. 2012). The empirical evidence shows otherwise.

How can it be that the desire to avoid pregnancy among women of reproductive age hardly changes? A first point is that the combined fractions of women who want to space or stop are high in most settings at most levels of fertility, typically exceeding 70 percent (see Table 2); with an arithmetic ceiling of 100 percent and an empirical ceiling probably on the order of 90 percent, the amount of change is constrained. SSA is an exception, with an average value for Variant B of 66 percent. The *Demand* construct does not suffer from the same arithmetic constraint. On the contrary, its features discussed above have the effect of obscuring the fundamental fact that at any given moment women in most reproductive regimes want to delay the next birth or have no further births. This is the case even at higher levels of fertility with the important exception of SSA societies.

Beyond this, there is a simple demographic explanation for the puzzling outcome: the parity distribution of in-union women has shifted toward lower parities and women at lower parities are less likely to want to terminate childbearing. This shift has been sufficient to largely offset increases in parity-specific desires to have no further children. This compositional shift in turn can be explained by later age at first birth (Bongaarts, Mensch, and Blanc 2017) and longer interbirth spacing (Casterline and Odden 2016; Timæus and Moultrie 2020), both of which have the effect of placing women at a lower parity at any given age. To our knowledge, this change in the demographic structure of reproductive-age women has not heretofore received attention in the research literature.

To demonstrate how changes in parity composition have worked against an increase in the prevalence of the desire to avoid pregnancy, we perform another demographic decomposition exercise, namely a decomposition of the change in fertility preference into contributions of change in parity composition (“composition” component) and change in parity-specific preferences (“rates” component). Table 6 summarizes the results, by region—desire to stop childbearing in the top panel, and the desire to delay or stop childbearing in the lower panel. For all regions, both measures change slowly over time—on average 0.7 percentage points per decade for the first measure and 0.1 percentage points per decade for the second measure (see rows “total change”). But had the parity composition remained constant, the average change in the two measures would have been decidedly larger – 2.7 and 0.5 percentage points, respectively (see rows “Parity-specific preferences”). By contrast, had only parity composition changed, and parity-specific preferences remained the same, the desire to delay or stop would have declined over time (see rows “Parity composition”). Note that the offsetting effect is stronger for the desire to stop than for the combined desire to delay or stop, as would be expected: the desire to stop childbearing is more closely tied to parity. And, the offsetting is larger in other regions than in SSA; in the other regions as compared to SSA, reproductive transitions have progressed further, including postponement of the first birth and lengthening of interbirth intervals.

**FIGURE 1:**
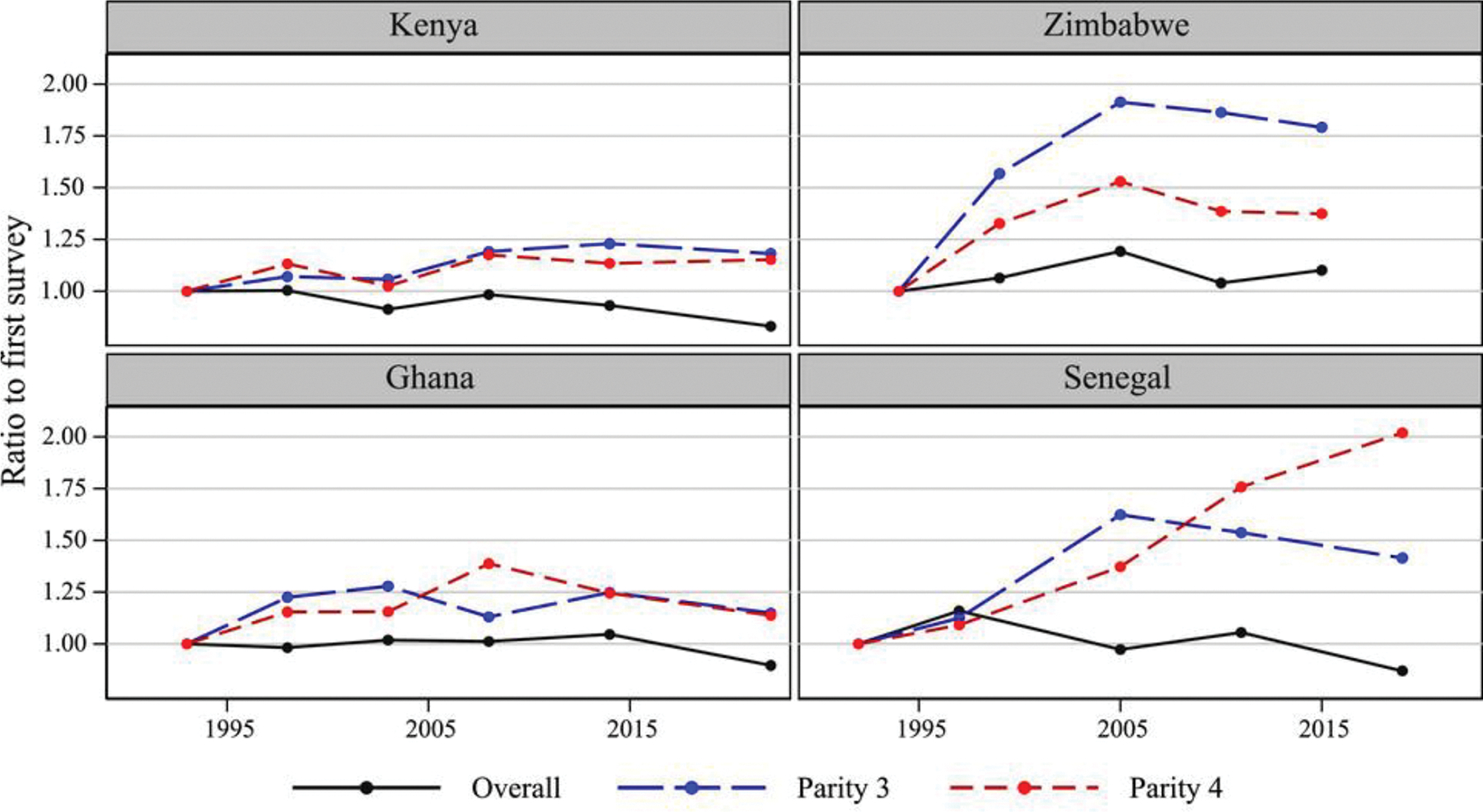
Trends in the desire to stop childbearing ratio to the first survey: Sub–Saharan Africa SOURCE: Survey data (DHS).

**FIGURE 2:**
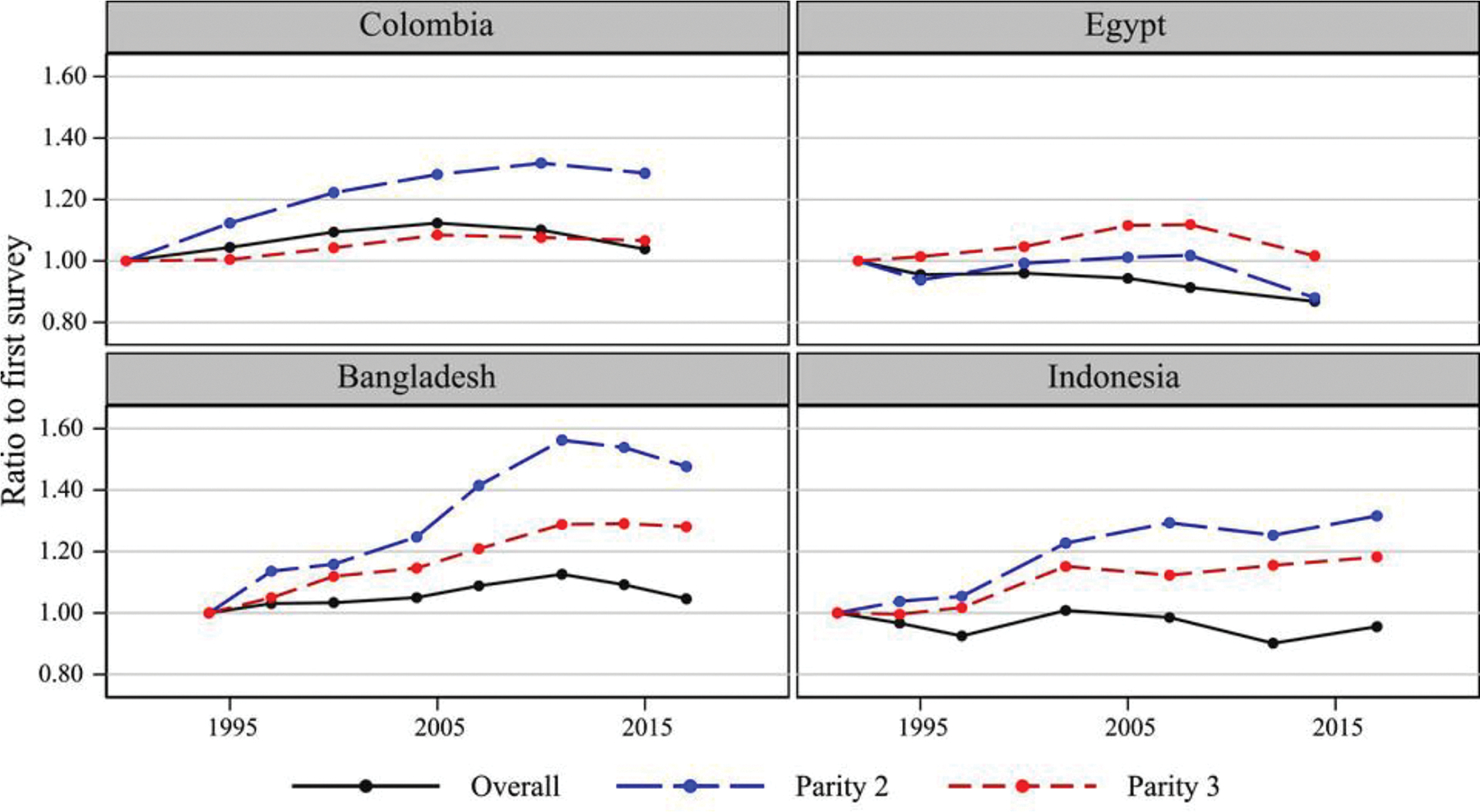
Trends in the desire to stop childbearing ratio to the first survey: other regions SOURCE: Survey data (DHS).

Figures 1 and 2 illustrate these results. Both figures show historical trends in the fraction of women who want to stop childbearing, expressed as a ratio to the first survey in each country. Lines are drawn for women of all parities pooled and parity-specific in eight selected countries (SSA in Figure 1; other regions in Figure 2). The contrast between the almost flat trend overall (women of all parities pooled) and the increase at middle parities (parities 3 and 4 in SSA, parities 2 and 3 in other regions) demonstrates that the flat overall trend disguises parity-specific increase in the desire to stop childbearing that is substantial in some countries (e.g., Bangladesh, Indonesia, Zimbabwe, Senegal). Shift in the parity composition toward lower parities has offset the impact of parity-specific preference change on the overall trend. But the latter change should not be slighted: parity-specific desires to have no further children have generally increased, a development entirely consistent with mainstream fertility transition theory. Had these parity-specific changes been far smaller, presumably the overall fraction of women desiring to stop would have declined in many countries.

## CONCLUDING COMMENTS

Trends in the desire to avoid pregnancy are of considerable interest for multiple reasons: They are central to major competing theories of fertility decline (Davis 1967; Pritchett 1994; Günther and Harttgen 2016; Bongaarts and Hodgson 2022), and they underlie key SRH indicators (most notably the SDG indicator “demand satisfied”) (Choi et al. 2015). National demographic survey data (DHS, MICS [Multiple Indicator Cluster Surveys]) offer several alternative indicators of the desire to avoid pregnancy, in particular, prospective fertility preferences (to delay or to stop) and the *Demand* construct (the sum of contraceptive prevalence and “unmet need for contraception”).

Through empirical analysis of survey data, we have shown that trends in fertility preferences and trends in *Demand* are substantially different, with the latter showing far steeper increases over time. Accordingly, decompositions of contraceptive change in the decades since 1970 give far larger credit to change in fertility desires, as against change in the implementation of desires, when desires are represented by the *Demand* construct. Indeed, demographic decomposition based on prospective preferences indicates that change in fertility desires has made a very small contribution; contraceptive change has been driven overwhelmingly by more complete implementation of fertility desires (i.e., change in the rates of contraceptive use within fertility preference categories). Note that the latter includes a small contribution from women who, judging from their stated preferences, do not desire to avoid pregnancy (Karra and Canning’s 2023: “unwanted contraception”). These differing results have implications for the credit assigned to the expansion of family planning services during the past five decades.

Why have trends in *Demand* been far steeper than trends in prospective fertility preferences? We have argued, with supporting empirical evidence, that trends in the *Demand* construct are in part a function of trends in contraception: an increase in contraception produces an increase in *Demand*, simply due to the construction of *Demand*. Hence *Demand* shows a historical increase that goes well beyond women’s measured attitudinal change; apparent change in fertility desires is an artifact of measurement. We do not assert that this renders the *Demand* construct invalid for all purposes. But it does make it a misleading basis for assessing trends in the desire to avoid pregnancy and for assessing the contribution of changes in fertility desires to contraceptive transition. More to the point, because *Demand* is in part a function of contraceptive prevalence, to rely on this construct in the decomposition analysis of contraceptive change is to fall victim to a form of “reverse causality” bias. In our empirical analysis, the resulting distortion is on the order of 25 percentage points (out of 100 percentage points in total).

We have also considered the puzzling empirical fact of slight change over time in the fraction of women who want to space or stop childbearing. We show that this is attributable simply to a shift in parity composition toward lower parities, itself due to the historical trend toward later age at first birth and lengthier interbirth spacing. When examined on a parity-specific basis, however, the fraction who want to space or stop has increased substantially in most countries. We do not wish to downplay the significance of this reproductive change, which conforms to standard expectations about the nature of fertility transition (Bongaarts 1992).

Our “cautionary note” is that assessments of trends in the desire to avoid pregnancy—and, by extension, demand for contraception—should be attentive to both measurement convention and demographic dynamics, as failure to do so can yield misleading conclusions. Additionally, the evidence presented in this note argues for revising the algorithms for unmet need (and accordingly *Demand*) so that users and nonusers are treated more consistently. Rather than a first division of the sample according to contraceptive use status, the unmet need algorithm should begin by ascertaining “need” on the basis of fertility preferences, adhering to the principle that “need for contraception” is conceptually a matter of fertility desires. The distinction between met need and unmet need would then be a function of contraceptive use status. Revision of *Demand* would follow automatically. This revision—which we believe is both a clarification and an improvement—would require no new survey items. Note that Canning and Karra (2023) make essentially the same recommendation.

It is important to recognize that *Demand* as conventionally measured and incorporated in SRH indicators, has other limitations that we have not addressed here. These limitations include the omission of women not in stable unions, the neglect of men’s desires, as well as the use of contraception for reasons other than limiting or spacing childbearing (to protect from sexually transmitted diseases, for instance). There is also an ongoing debate among scholars and activists regarding the appropriateness, if one adopts a reproductive-rights-based approach, of a focus on “need” rather than on individuals’ wants and desires, including satisfaction with their contraceptive method (Holt et al. 2023; Rominski and Stephenson 2019).

Such critique notwithstanding, the conventional *Demand* construct remains a widely used indicator that in our view is valid for the limited (but important) purpose for which it was devised. Moving beyond this now well-established construct, successfully addressing the issues highlighted above would yield new indicators that may prove more informative both for social scientists striving to understand reproductive change and for the purpose of policy formulation and program planning. We are encouraged by the emerging literature replete with proposals about how these various issues might be addressed via new analytic approaches and/or new survey items (Karra 2022; Rothschild, Brown, and Drake 2021; Senderowicz and Maloney 2022; Sinai, Igras, and Lundgren 2017; Bornstein et al. 2023; Senderowicz et al. 2023). We caution that the temptation to infer attitude from behavior no doubt will remain, with the attendant risk of “reverse causality” of the sort that we have identified and analyzed in this research. That is to say, the danger of distorted assessment of trends in demand for contraception, however defined and measured, will persist. Ultimately this is a matter of conceptual clarity: if demand for contraception and contraceptive behavior are conceptualized as distinct phenomena, then the respective indicators should be independent in their construction. We have shown how the conventional *Demand* construct fails this test.

## Figures and Tables

**TABLE 1:** Measures of the desire to avoid pregnancy (yes/no)

		Desire to avoid pregnancy variant				
Input measures	A	B	C	D	E	F

Preferences:^a^ currently pregnant	Prospective	Retrospective	Retrospective	Retrospective	Retrospective	Retrospective
Preferences:^b^ postpartum amenorrheic	Prospective	Prospective	Retrospective	Retrospective	Users = Prospective / Nonusers = Retrospective	Users = Prospective / Nonusers = Retrospective
Infecundity^c^	Preference item	Preference item	Preference item	Unmet need item	Unmet need item	Unmet need item
Women who “want soon”^d^	No desire	No desire	No desire	No desire	No desire	*Demand*

a “Prospective”: preference for another birth after the current pregnancy. “Retrospective”: wantedness status of current pregnancy.

b “Prospective”: desire for future birth. “Retrospective”: wantedness status of last birth. “Users/nonusers”: identified according to the current use of any contraceptive method.

c “Preference item”: infecund if answers “can’t get pregnant” to the preference question. “Unmet need item”: multiple indicators, including never users with no pregnancy within the last five years. All users are considered fecund.

d “No desire”: if “want soon” according to the prospective preferences or retrospective wantedness of pregnancy/birth, classified as not desiring to avoid pregnancy. “*Demand*”: “want soon” women have no desire if not using contraception but have a desire for spacing if using.

**TABLE 2:** Percentage of women who desire to avoid pregnancy: Six measures (mean survey value, by region)

Desire to avoid pregnancy variant^a^	Sub-Saharan Africa	Other regions	All regions

A. Prospective preferences entirely	74.1	84.7	78.5
B. Prospective preferences, except currently pregnant	65.6	79.3	71.3
C. Prospective preferences, except post-partUm amenorrheic and currently pregnant	51.4	73.5	60.7
D. As DHS unmet need for infecund	47.3	69.5	56.6
E. As DHS unmet need for infecund and post-partum amenorrheic	49.3	71.1	58.4
F. *Demand* - DHS unmet need + all users	52.5	74.5	61.8
Number of surveys	133	96	229
Number of countries^b^	33	20	53

NOTE: Sample, currently in-union women aged 15–44.

a See Table 1.

b Countries with two or more surveys. DHS versions 2–8 (1990–2022).

**TABLE 3:** Trends in the percentage of women who desire to avoid pregnancy (average percentage point increase per decade: Linear regression estimate^a^)

Desire to avoid pregnancyvariant^b^	Sub-Saharan Africa	Other regions	All regions

A. Prospective preferences entirely	0.8	−0.4	0.3
B. Prospective preferences, except currently pregnant	1.4	−0.2	0.7
C. Prospective preferences, except post-partum amenorrheic and currently pregnant	2.8	1.2	2.2
D. As DHS unmet need for infecund	3.0	2.1	2.6
E. As DHS unmet need for infecund and post-partum amenorrheic	3.8	2.3	3.2
F. *Demand*—DHS unmet need + all users	5.0	3.0	4.2
Number of countries^c^	33	20	53

NOTE: Sample, currently in-union women ages 15–44.

a Slopes on year (expressed per decade) from linear regression with a fixed effect for country.

b See Table 1.

c Countries with two or more surveys. DHS versions 2—8 (1990–2022).

**TABLE 4:** Decomposition of contraceptive change: Composition component (median percentage contributed by change in preferences / *Demand*)

Desire to avoid pregnancy variant^a^	Sub-Saharan Africa	Other regions	All regions

B. Prospective preferences, except currently pregnant	4%	0%	3%
C. Prospective preferences, except post-partum amenorrheic and currently pregnant	8%	9%	8%
D. As DHS unmet need for infecund	12%	20%	13%
E. As DHS unmet need for infecund and post-partum amenorrheic	17%	16%	17%
F. *Demand* - DHS unmet need + all users	28%	29%	28%
Number of decompositions^b^	37	28	65
Number of countries	26	18	44

NOTE: Sample, DHS surveys, currently in-union women ages 15–44.

a See Table 1.

b Selection criteria: surveys spaced at least 6 years apart and change in Contraceptive Prevalence Rate at least 6 percentage points.

**TABLE 5:** Assessing sources of contraceptive change: Fictional illustration

	First survey		Second survey	
Preference category	Preference composition	Percent using	Preference composition	Percent using

Want soon	0.25	6	0.25	16
Want later	0.35	40	0.35	55
Want no more	0.40	65	0.40	75
Total	1.00	41	1.00	53
*Demand*	76		79	
*Demand Satisfied*	54		67	
Decomposition Approach				
(1) Conventional demographic: Percentage of composition		0		
(2) *Demand - Demand Satisfied*: Percentage of *Demand*		12		

**TABLE 6:** Decomposition of preference change: Percentage points per decade (mean percentage points contributed by parity composition and by parity-specific preferences)

Type of preference^a^ and decomposition component	Sub-Saharan Africa	Other regions	All regions

Desire to stop			
Parity composition	−1.2	−3.4	−2.0
Parity-specific preferences	2.7	2.6	2.7
Total change	1.5	−0.8	0.7
Desire to delay or stop			
Parity composition	−0.2	−0.9	−0.4
Parity-specific preferences	0.7	0.2	0.5
Total change	0.5	−0.7	0.1
Number of decompositions^b^	69	40	109
Number of countries	33	18	51

a Prospective preferences entirely (Variant A).

b Selection criteria: surveys spaced at least 6 years apart.

## Footnotes

1 Variant A is excluded because contraceptive use status for currently pregnant women according to their prospective preference cannot be assessed, as it refers to the future after they give birth.

2 For examples of such decompositions, see Feyisetan and Casterline (2000) and Ibitoye, Casterline, and Zhang (2022). Both analyses use Variant B.

3 As indicated in Table 4, 44 countries contribute to the decomposition exercise. The selection rules are (i) surveys must be at least six years apart; (ii) Contraceptive Prevalence Rate must have increased by at least 6 percentage points. These two conditions guarantee that enough time has elapsed and enough change has occurred to merit performing a stable change decomposition, without drastically cutting down the sample size of countries and surveys. The results are not sensitive to variations in the selection criteria according to sensitivity analyses that we have carried out (e.g., selection rules of 10 years and 5 percentage points).

4 Presumably Bongaarts and Hodgson carry out one decomposition per country. They do not report the number of countries.

5 Because of this treatment of “want soon” women, decompositions based on *Demand* and *Demand Satisfied* (e.g., Bongaarts and Hodgson 2022) are strictly speaking not conventional demographic decompositions (as explained, e.g., in Kitigawa (1955) and Das Gupta (1993)). The composition and rates contributions are confused.

6 In this fictional illustration, 2.5 percentage points out of the total 12 percentage point increase in contraception is contributed by “want soon” women. This is in accordance with DHS data: in most every country and time-period, increased use among this subgroup constitutes 10–20% of the contraception increase (results available from the authors).

7 In the current DHS unmet need algorithm, infecund status is ascertained using multiple indicators, six in total. One of the six indicators is “can’t get pregnant” response to the fertility preference item; these women’s fertility preferences are unknown, and hence they cannot be classified as having either need or no need. The other five indicators are another matter. Two indicators presume no use of contraception (current or past), while the remaining three are based on responses to the recency of menstruation item. Ceteris paribus secular increases in contraception can be expected to reduce the fraction of women classified as infecund according to one or more of these five indicators.

8 We thank a reviewer for drawing our attention to this point.
